# A two-stage genome-wide association study of radiation-induced acute toxicity in head and neck cancer

**DOI:** 10.1186/s12967-021-03145-1

**Published:** 2021-11-27

**Authors:** Elnaz Naderi, Anne Petra Gerarda Crijns, Roel Johannes Henricus Marinus Steenbakkers, Johanna Geertruida Maria van den Hoek, Hendrika Marike Boezen, Behrooz Ziad Alizadeh, Johannes Albertus Langendijk

**Affiliations:** 1 Department of Radiation Oncology, University Medical Center Groningen, Hanzeplein 1, HPC; DA 30, P.O. Box 30 001, 9700 RB Groningen, The Netherlands; 2 Department of Epidemiology, University Medical Center Groningen, Groningen, The Netherlands

**Keywords:** Radiogenomics, GWAS, Radiation-induced toxicity, Head and neck cancer

## Abstract

**Background:**

Most head and neck cancer (HNC) patients receive radiotherapy (RT) and develop toxicities. This genome-wide association study (GWAS) was designed to identify single nucleotide polymorphisms (SNPs) associated with common acute radiation-induced toxicities (RITs) in an HNC cohort.

**Methods:**

A two-stage GWAS was performed in 1279 HNC patients treated with RT and prospectively scored for mucositis, xerostomia, sticky saliva, and dysphagia. The area under the curve (AUC) was used to estimate the average load of toxicity during RT. At the discovery study, multivariate linear regression was used in 957 patients, and the top-ranking SNPs were tested in 322 independent replication cohort. Next, the discovery and the replication studies were meta-analyzed.

**Results:**

A region on 5q21.3 containing 16 SNPs showed genome-wide (GW) significance association at P-value < 5.0 × 10^–8^ with patient-rated acute xerostomia in the discovery study. The top signal was rs35542 with an adjusted effect size of 0.17*A (95% CI 0.12 to 0.23; P-value <  = 3.78 × 10^–9^). The genome wide significant SNPs were located within three genes (*EFNA5, FBXL17,* and *FER*). *In-silico* functional analysis showed these genes may be involved in DNA damage response and co-expressed in minor salivary glands. We found 428 suggestive SNPs (P-value < 1.0 × 10^–5^) for other toxicities, taken to the replication study. Eleven of them showed a nominal association (P-value < 0.05).

**Conclusions:**

This GWAS suggested novel SNPs for patient-rated acute xerostomia in HNC patients. If validated, these SNPs and their related functional pathways could lead to a predictive assay to identify sensitive patients to radiation, which may eventually allow a more individualized RT treatment.

**Supplementary Information:**

The online version contains supplementary material available at 10.1186/s12967-021-03145-1.

## Background

Head and neck cancer (HNC) is a life-threatening disease affecting approximately 650,000 new patients and causing 330,000 deaths annually worldwide [1]. More than 90% of HNC are squamous cell carcinomas affecting the mucosal membranes, salivary glands, swallowing muscles, craniofacial bones, and soft tissues.

Curative treatment modalities for HNC include surgery, radiotherapy (RT), chemotherapy, targeted agents, or a combination of these depending on the primary tumour site and locoregional tumour extension [2]. Almost 80% of HNC patients receive RT at some stage of their treatment [3]. The main aim of RT is to obtain long-term tumour control while minimizing healthy tissue damage. However, RT is associated with collateral damage to the healthy tissues surrounding the tumour, resulting in a broad spectrum of acute and late radiation-induced toxicities (RITs) [4]. Patients receiving RT experience varying levels of toxicity, from minor to severe for a period of a few weeks to even lifetime, hampering patients’ quality of life [5, 6], such as mucosal inflammation (mucositis), mucosal infections, sticky saliva, dry mouth (xerostomia), chronic pain, decreased voice quality, impaired chewing and swallowing (dysphagia).

The risk of RIT is assessed using normal tissue complication probability (NTCP) models. NTCPs are multivariable prediction models built on radiation dose metrics, clinical factors, and patients’ characteristics [7]. The performance of current NTCP-models is suboptimal due to patients’ differences in normal tissue radio-sensitivity, determined by underlying genetic susceptibility [8, 9]. Initial evidence suggests individuals affected by ataxia-telangiectasia and Nijmegen Breakage Syndrome, two genetic disorders, are hypersensitive to radiation and unable to undergo RT [10]. Nevertheless, the heritability of radio-sensitivity has not been thoroughly investigated. Some studies based on flow cytometric assays estimated a heritability of 58 to 78% for cell response to irradiation, which was, in turn, a proxy for radio sensitivity [11–13]. Likewise, two previous studies showed associations between single nucleotide polymorphisms (SNPs) in *XRCC1*, *RAD51,* and *NBN* genes with the development of grade ≥ 2 radiation-induced mucositis, dysphagia, and skin erythema in HNC patients [14, 15]. Recently, several genome-wide association studies (GWAS) have identified SNPs associated with RITs in breast and prostate cancer patients [8, 16]. For HNC, a recent GWAS observed significant associations between three SNPs on chromosome 5 with acute mucositis (Line M H Schack et al. under review). Another GWAS found 50 suggestive loci associated with mucositis in Chinese HNC patients [17]. The underlying genetic causes of RITs remain undiscovered yet. We performed a two-stage GWAS in a Dutch HNC cohort to identify SNPs and subsequent potential genetic pathways associated with acute RITs.

## Methods

An extended description of the methods and results can be found in the Additional file 1: Appendix.

### Study design

We performed a two-stage GWAS. At discovery study, we aimed to identify candidate SNPs associated with acute RIT in HNC patients. At the replication study, we sought to confirm the identified discovery SNPs in independent HNC cohort. To gain more statistical power for discovery, we combined discovery and replication cohorts and performed a GWAS for acute RIT (i.e., combined analysis).

### Participants

We included 1429 HNC patients (1102 for the discovery; 327 for the replication study). Patients were treated with primary or postoperative RT either with or without chemotherapy from 2007 to 2020 as part of the UMCG-HNC prospective data registration program (NCT02435576, clinicaltrials.gov). The prospective data registration program has been reviewed by the medical ethical committee and is considered standard of care. Additional written informed consent was obtained for the genetic study (NCT02489084). Data on baseline patient-, tumour-, and treatment- characteristics were collected before starting RT. Additionally, physician and patient-rated acute RITs were prospectively assessed weekly during RT and after completion of RT, up to 7 weeks (called acute RITs)[18].

### Assessment of RITs

Physician-rated acute toxicities, including mucositis, dysphagia, xerostomia, and sticky saliva, were registered according to the Common Toxicity Criteria of Adverse Events (version 4.0) [19]. Patient-rated HNC symptoms were assessed using the EORTC QLQ-H&N35 questionnaire in addition to the EORTC QLQ-C30 [20] (Additional file 1: Table S1).

### Multiple imputations of missing value for toxicity

We observed varying percentages of missingness across RITs (Additional file 1: Table S2). We applied multiple imputation (MI) as implemented in MICE package [21] (Additional file 1: Methods).

### Genotyping, quality control, and imputation

Samples were genotyped using the Illumina human core and GSA arrays. We applied standard participant and SNP level quality control (QC). We checked the ethnicity of participant using multidimensional scaling clustering using EIGENSTRAT. Missing genotypes were imputed based on the HRC reference panel (Additional file 1: Supplementary Methods).

### Clinical factors

Based on clinical relevance confirmed by a panel of experts of the Radiogenomics Consortium[22], age, gender, type of RT, concomitant chemotherapy, tumour-site, and baseline toxicity were included as co-variables (Table 1). We added the volume surrogate as defined by Volume 1 = T1a − 1bN0M0 glottic laryngeal carcinomas, Volume 2 = all other TxN0 sites, and Volume 3 = TxN1-3 carcinomas (Line M H Schack et al. under review).

**Table 1:** Baseline characteristics of included HNC patients

Overall N	Discovery N = 957	Replication N = 322	Total N = 1279
Period of treatment	2007–2018	2018–2020	2007–2020
Gender; Female (%)	313 (32.71)	98(30.40)	411(32.10)
Mean age (SD)	63.50 (11.75)	65.70(11.63)	63.90 (11.75)
Age group (%)			
≥ 55	209 (21.84)	58(18.00)	267(20.90)
56–69	455 (47.54)	131(40.70)*	586(45.80)
70 ≥	293 (30.62)	133(41.30)***	426(33.30)
Tumor site (%)			
Oral cavity	180 (18.95)	47(14.70)	227(17.70)
Oropharynx	194 (20.42)	80(25.10)	274(21.40)
Larynx	256 (26.95)	82(25.70)	338(26.40)
Others	320 (33.68)	110(34.50)	430(33.60)
T-stage; T3, 4 (%)	362 (37.83)	155(51.70)	517(40.42)
N-stage; N1–3 (%)	456 (47.65)	154(51.70)	610(47.49)
Chemotherapy (%)			
No chemotherapy	691 (73.98)	222(71.20)	913(71.4)
Concomitant chemo	227 (24.30)	79(25.30)	306(23.90)
Radio + cetuximab	16 ( 1.72)	11(3.50)	27(2.10)
Radiotherapy			
Postoperative (%)	409 (42.74)	115(36.30)	524(40.97)
Volume Surrogate			
Glottic/laryngeal T1N0M0	123 (13.57)	25(8.60)*	148(11.60)
All other TxN0 sites	329 (36.32)	111(38.30)	440(34.40)
TxN1-3 carcinomas	454 (50.11)	154(53.10)	608(47.50)

*Significance difference between discovery and replication cohorts: *P < 0.05; **P < 0.01; ***P < 0.001

## Data analysis

### Outcome modelling

We assessed RITs using two scoring systems. First, since acute RIT generally increases during treatment, we estimated the area under the curve (AUC) to generate an overall measure of acute toxicity during RT treatment up to seven weeks per each of the RITs (Additional file 1: Supplementary methods). A mean of AUCs represents an average of toxicity per week during the RT plan. Second, to achieve a composite score representing the overall acute RIT, we used the standardized total average toxicity (STAT) [23]. STAT_*physician*_ and STAT_*patient*_ included the spectrum of the physician and patient-rated acute RITs, respectively.

### Association analyses

Multivariate linear regression was used to estimate the association of the additive effect of SNPs effect allele with AUCs and STAT scores. Multivariable models were adjusted for the aforementioned covariates and the top 10 PCA eigenvectors. SNPs were included as the number or dosage of effect alleles in (imputed) genotypes resulting in a regression coefficient for one copy increase in effect allele. A genome-wide P-value < 5.0 × 10^–8^ was considered statistically genome-wide significant (GWS), and a P-value < 1.0 × 10^–5^ was considered a suggestive association.

### Replication study and meta-analysis

The SNPs with a suggestive association identified in the discovery study were included in the replication study. We used the same co-variables. Each suggestive SNP was tested for the association with its corresponding RIT. A Bonferroni corrected P-value at 0.05/number of independent loci was considered as statistically significant replication. Per each SNP, the summary statistics of discovery and replication studies were meta-analysed using an inverse variance-weighted fixed-effect model implemented in METAL (version 2011-03-25) [24]. Any SNP with a meta-P-value of 1.0 × 10^–5^ >  < 5.0 × 10^–8^ was considered as a suggestive SNP and with a meta-P-value < 5.0 × 10^–8^ as replicated GWS SNP.

### Combined study

We combined discovery and replication cohorts (1,429 HNC patients) performed a GWAS for acute RIT to gain more statistical power for discovery.

### Power analysis

Quanto software [25] was used to estimate the study's statistical power. The study had 80% power to detect SNPs with effect allele frequencies > 0.45 and with an effect size of 2.0 or higher at P-value < 5.0 × 10^–8^.

### In-silico functional analysis

To understand the functional effects of the identified genome-wide associated SNPs in the discovery study (GWS_*discovery*_ SNPs), we performed an in-silico functional analysis. First, we used Ensembl [26] (release 98) to extract characteristics of the GWS_*discovery*_ SNPs. Next, we used SCREEN [27] to explore if the GWS_*discovery*_ SNPs compose cis-Regulatory Elements (ccREs), which regulate the expression of nearby genes. Then, we investigated whether ccREs were related to functional elements. We selected genes associated with ccREs and GWS_*discovery*_ SNPs using GeneCards (for biological function) and MalaCards (for related diseases) [28]. We performed gene expression analyses using GTEx (v.8) [29] to understand the expression levels of these genes across all tissues. Genes and tissues were clustered using hierarchical clustering, and eventually, tissues with similarity in expression of genes were clustered together.

## Results

### Patient characteristics

**Fig. 1:**
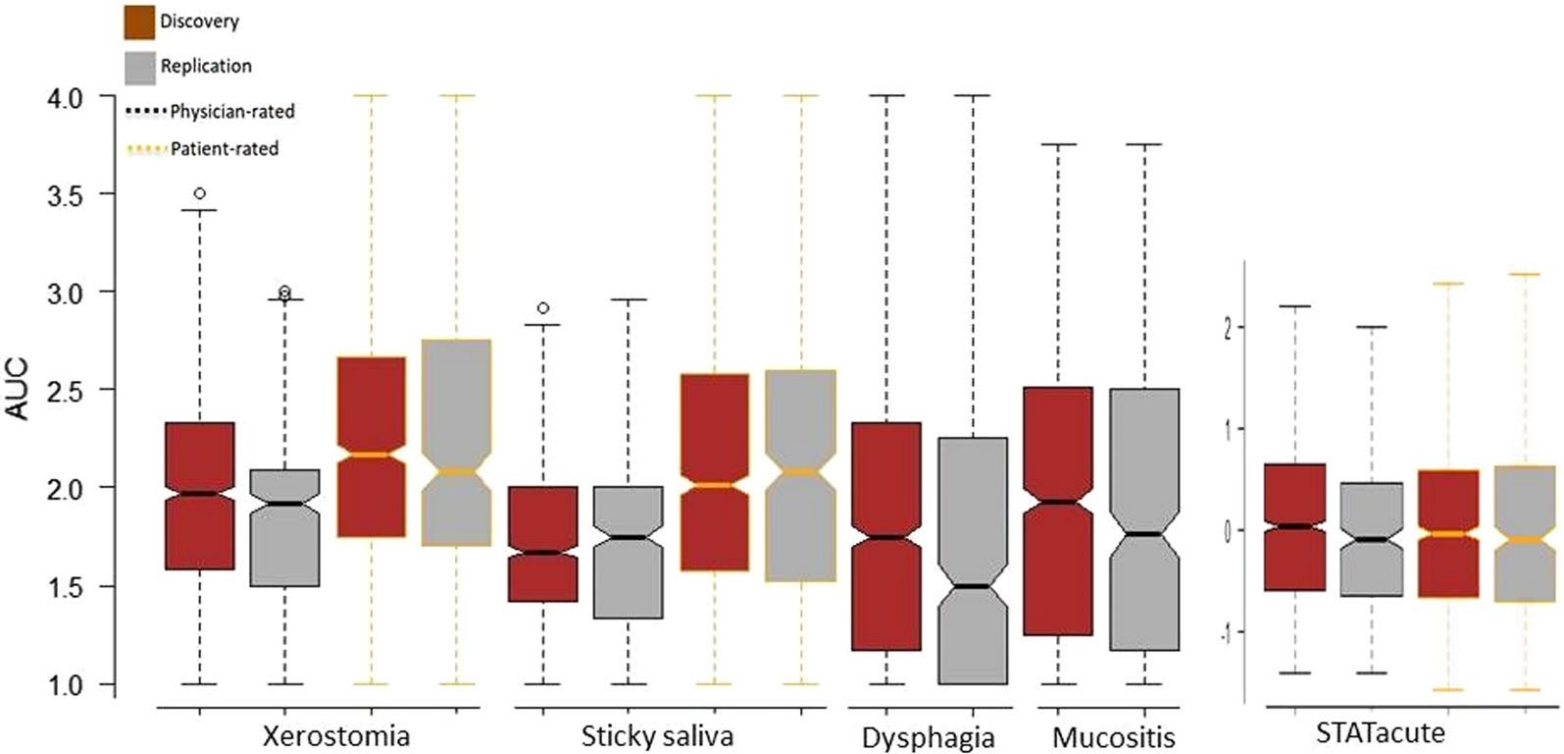
The dispersion of AUCs and STAT scores of acute radiotoxicity endpoints in HNC patients by discovery and replication cohorts. The X-axis shows the endpoints, and Y-axes show the measured value of the AUC and STAT score of endpoints. The Brown color represents the discovery study, and the grey color represents the replication study. The black border shows physician-rated endpoints, and the orange border shows patient-rated endpoints. The lowest line represents the minimum (Q0 or 0th percentile): the top line represents the maximum (Q4 or 100th percentile) data point excluding any outliers; The middle line represents the median (Q2 or 50th percentile); the box represents the interquartile range (IQR) which is the distance between the first quartile (Q1 or 25th percentile; that is the median of the lower half of the dataset) and the third quartile (Q3 or 75th percentile that is the median of the upper half of the dataset). Table 2 shows the comparison between discovery and replication studies for the significant difference in outcomes’ distribution

Table 1 describes the baseline and clinical characteristics of the included patients. Among 1429 patients with genotyped data, 35 patients were excluded due to QC and 115 patients did not have clinical data. In total, 1279 HNC patients (mean age 63.9 (SD ± 11.75) years being 67.9% men) were included in GWAS (Additional file 1: Fig. S2). We obtained 6,334,207 imputed SNPs for 957 HNC patients in the discovery study and 6,563,883 SNPs in 322 HNC patients in the replication study (Additional file 1: Fig. S2 & Table S4). Figure 1 and Table 2 describe the distribution of acute RITs in the discovery and replication studies. Additional file 1: Table S5 presents the association of predictors with each of the acute RITs.

**Table 2:** Average score (± SD) of RITs measure by the area under curve^a^ and STAT acute

	Discovery study	Replication study
AUC of physician-rated xerostomia	1.93 (± 0.51)	1.84 (± 0.50)^2^*
AUC of physician-rated sticky saliva	1.68 (± 0.43)	1.73 (± 0.49)
AUC of physician-rated dysphagia	1.89 (± 0.81)	1.73 (± 0.81)^2^*
AUC of physician-rated mucositis	1.95 (± 0.70)	1.90 (± 0.76)
STAT_physician_	0.02 (± 0.78)	− 0.06 (± 0.78)
AUC of patient-rated xerostomia	2.20 (± 0.71)	2.21 (± 0.77)
AUC of patient-rated sticky saliva	2.08 (± 0.72)	2.13 (± 0.78)
STAT_patient_	0.00 (± 0.87)	0.02 (± 0.96)

*AUC* area under curve; *STAT* standardized total average toxicity

^a^Fig. 1 shows details of the distribution of outcomes

*Significance difference between discovery and replication cohorts: *P < 0.05 or ^2^*P < 0.01

### GWAS

We found sixteen GWS_*discovery*_ SNPs, tagged one locus, associated with patient-rated acute xerostomia. The 428 GW suggestive SNPs spanned over 117 loci associated with acute RITs (Additional file 1: Fig. S3). Neither the 16 GWS_*discovery*_ SNPs, nor the 428 GW suggestive SNPs were significantly associated with their corresponding endpoints in the replication study (Additional file 2: Table S6). Eleven out suggestive SNPs showed a nominal association to their corresponding acute RITs (Additional file 2: Tables S7–S14). We found no SNP was significantly associated with any of the eight tested acute RITs in combined analysis. There were 710 GW suggestive SNPs spanned across 137 genomic regions associated with different acute RITs (Additional file 1: Fig. S3).

### Patient-rated acute xerostomia

**Fig. 2:**
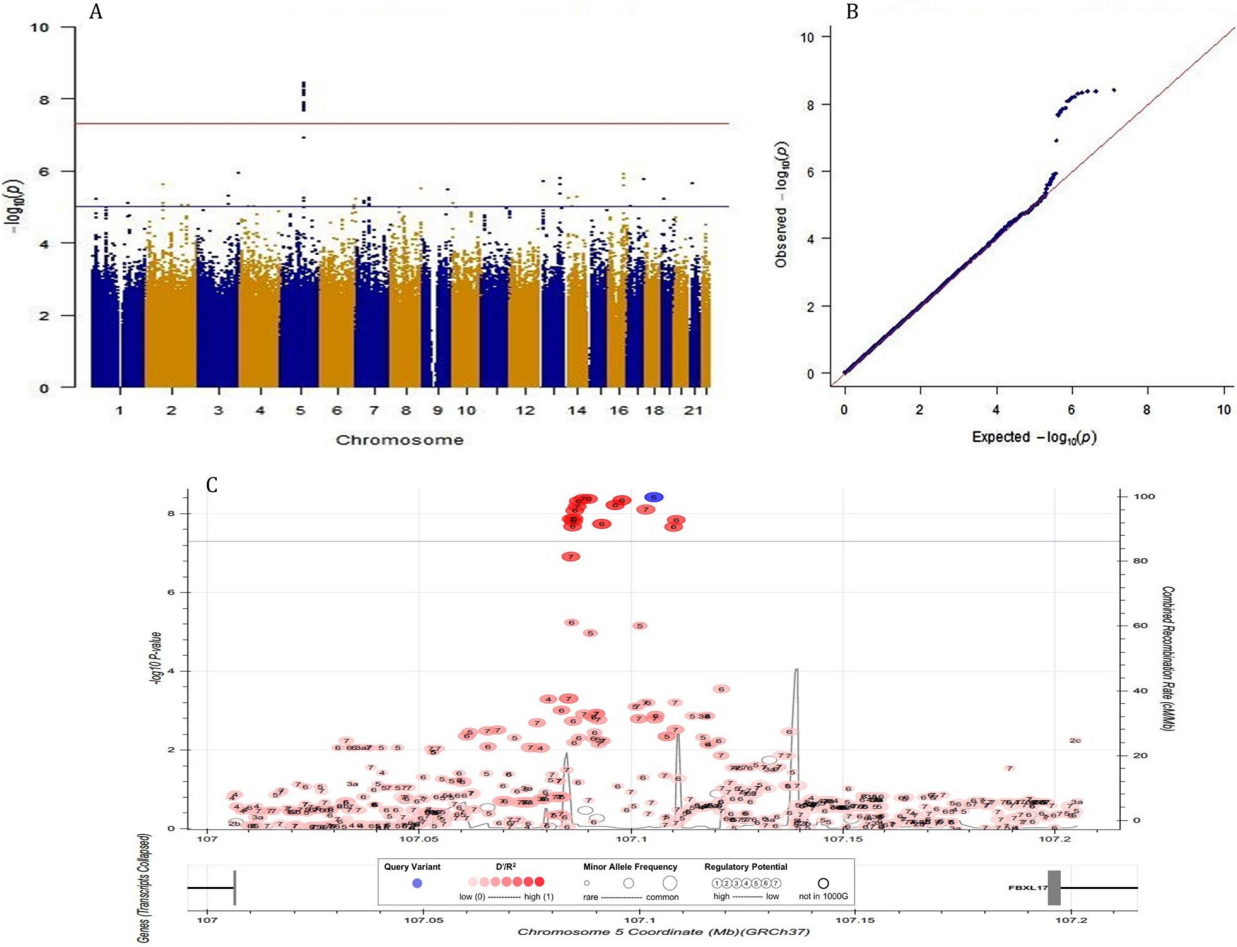
Genome-wide association findings for AUC for patient-rated toxicity xerostomia in HNC patients. 2A. Manhattan plot: The X-axis shows the location in the genome. The Y-axis shows − log10 P-values for the association of each of the tested SNPs with the outcome. The red line shows the threshold for genome-wide significance (P < 5 × 10–8), and the blue line shows the suggestive threshold (P < 1 × 10–5). 2B. Quantile–quantile (QQ) plot comparing the distribution of observed P-values (test statistics) from discovery study to the distribution of expected P-value based on the theoretical probability distribution, inflation of plot to upper part suggest inflation of test statistics due to the possibility of population substructure or type 1 error (small sample size bias). The Y-axis shows observed − log10 P-values, and the X-axis shows the expected − log10 P-values. Each SNP is plotted as a dark blue dot, and the red line indicates a null hypothesis of no true association. Deviation from the expected P-value distribution is evident only in the tail area, with a lambda of 1.001, suggesting that population stratification was adequately controlled. 2C. Locuszoom plot of the associated region on chromosome 5. The blue circle (query variant) points to the top SNP (rs35542). Points representing nearby SNPs are color-coded according to linkage disequilibrium r2 value as indicated in the legends. The X-axis shows the genomic coordinates chromosome 5. The Y1 axis shows − log10 P-values for each of the SNPs in the genome. The Y2 axis shows the combined recombination rate which is estimated from the international HapMap project

Among the 16 GWS_*discovery*_ SNPs associated with patient-rated acute xerostomia in the discovery study (Fig. 2A, B), the top signal was rs35542 with an association effect size (beta) of 0.17 (95% CI 0.12 to 0.23; P-value ≤ 3.78 × 10^–9^) per increase one copy of A effect allele. These 16 SNPs were in high linkage disequilibrium (LD) (Additional file 1: Fig. S4). Additionally, 43 SNPs from 15 genomic regions were suggestively (P-value < 1.0 × 10^–5^) associated with patient-rated acute xerostomia. None of the identified GWS_*discovery*_ SNPs or suggestive SNPs reached into a statistically significant replication P-value. For the top rs35542 SNP, the meta-analysis reached a meta-effect size of 0.13 (95% CI 0.08 to 0.18; P_*meta*_ = 5.31 × 10^–7^ (Additional file 2: Table S7).

Additional file 1: Supplementary result and Additional file 2: Tables S7–S14 report the details of variants with a suggestive associations at P-value < 1.0 × 10^–5^ with all endpoints.

### In-silico functional analysis

**Fig. 3:**
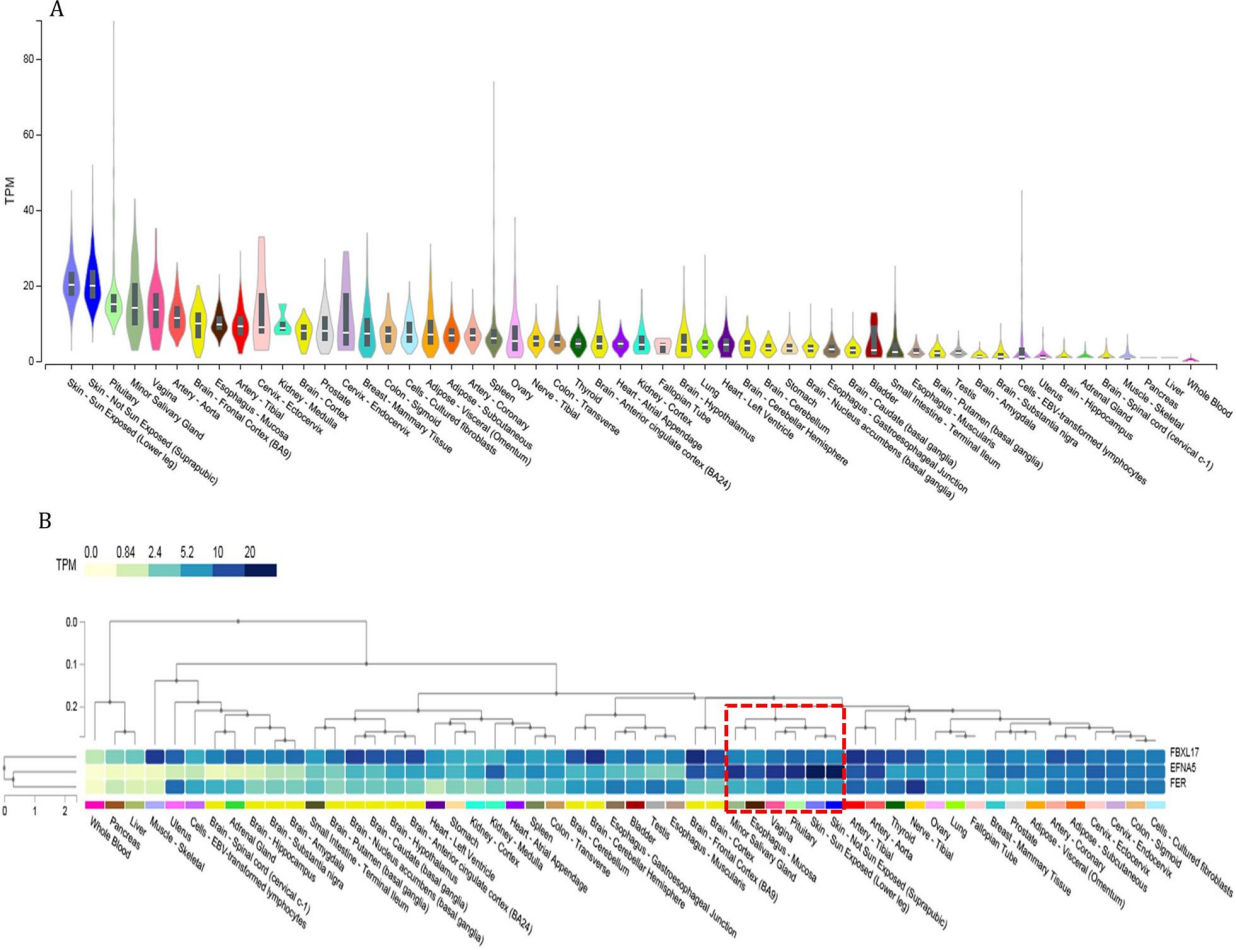
Single and multi-gene expression visualization across all tissues, obtained from GTEx. 3A: Single expression visualization of EFNA5 gene, as the nearest gene, is shown. The Y-axis shows the expression density of EFNA5 measured as linear count of transcript per million (TPM), and the X-axis shows the tissues sorted as a decrease in the median of TPM. Box plots are shown as median and 25th and 75th percentiles; points are displayed as outliers if they are above or below 1.5 times the interquartile range. 3B: A heat map of multi-gene expression visualization for EFNA5, FBXL17, and FER genes is shown. The genes and tissues are clustered using hierarchical clustering. The expression level of the gene per tissue is color-coded according to the linear count of TPM as indicated by the legend. The red box shows sub-clusters of high expression levels of the three genes in secretory tissues, including the minor salivary gland

Annotation analysis showed the GWS_*discovery*_ SNPs with patient-rated acute xerostomia were mapped into a noncoding genomic-block (107085963 to 107110731 base pair; GRCh37.p13.chr5) homing to ccREs identified by ENCODE functional dataset. The ccREs were consistently subjected epigenetic activity by histone modification of the H3K27Ac (acylation of lysine 27 of histone 3) and H3K4me3 (methylation of lysine 4 of histone 3). The Ephrin-A5 (*EFNA5),* F-Box and Leucine-Rich Repeat Protein 17 (*FBXL17),* and FER Tyrosine Kinase (*FER)* genes are co-localized on the same block with these ccREs, where the GWS_*discovery*_ SNPs reside (Fig. 2C). Using GeneCards and MalaCards, showed *EFNA5, FBXL17,* and *FER* have been associated with severe combined immunodeficiency characterized by sensitivity to ionizing radiation disease, DNA damage response after ionizing radiation and activation of the ataxia-telangiectasia mutated protein, respectively. The GTEx tissue-specific expression profiles showed the nearest gene, *EFNA5*, is highly expressed in minor salivary glands with a median expression level equal to 14.04 TPM (Fig. 3A). Using multi-gene query visualization showed these three genes have the same co-expression pattern in secretory tissues including the minor salivary gland, vagina, and pituitary and also in sun-exposed skin (Fig. 3B).

## Discussion

This GWAS aimed to identify SNPs associated with acute RITs in HNC patients. In the discovery study, we found 16 SNPs associated with patient-rated acute xerostomia at the GWS level. The functional analysis showed plausible biological mechanisms supporting that identified GWS SNPs may play a role in radiation response in healthy tissues generally. However by replication and meta-analysis, our top significant hits shifted up to the suggestive associations. Also, 428 SNPs showed suggestive association with other acute RITs. The majority showed consistent effect directions in discovery and replication studies. By combined analysis, none of the SNPs was GWS, which is likely due to type II error lack of statistical power (i.e. false negative). By combined analysis, we found more suggestive SNPs (710 in 137 loci) associated with acute RITs than two-staged GWAS analysis.

We found a genomic block on 5q21.3 consisting of 16 highly linked (in LD) SNPs associated with patient-rated acute xerostomia at the discovery study. The only concurrent GWAS in HNC was by Schack et al. who observed significant associations between three SNPs mapped on chromosome 5(5q31.2) with acute mucositis (Line M H Schack et al. under review). There is no other GWAS analysis of HNC patients in Caucasians. Although a Chinese GWAS found 50 suggestive genomic loci (P-value < 1.0 × 10–4) mapped in 65 genes associated with radiation-induced mucositis in HNC patients [17], but we failed to replicate their findings in our study. However, in breast cancer by using a 2-stage design of 305 SNPs across 59 candidate genes, Seibold et al. reported the association of rs2682585 SNP in the base excision repair gene *XRCC1*, with late RIT in breast cancer [30]. In a recent meta-analysis of European ancestry cohorts, Kerns et al. identified three SNPs associated with rectal bleeding, decreased urinary stream, and haematuria after RT for prostate cancer [31]. A meta-analysis showed a significant association between the ataxia telangiectasia mutated (*ATM)* rs1801516*Asn allele with increased risk of radiation-induced tissue toxicity in breast and prostate cancers [16]. Though there were no other GWAs in HNC to be compared, the ATM associated pathway in two cancers seems interestingly linked to the functional analysis of annotation of the 16 GWS_discovery_ SNPs. These SNPs located across several ccREs bounded to H3K4me3 (methylation of lysine 4 of histone 3). H3K4me3 is implicated in repairing of double-strand DNA breaks (DSBs) caused by ionizing radiation during RT [32, 33]. Furthermore, the neighbouring genes, including *EFNA5, FBXL17,* and *FER,* are related to radio-sensitivity*. EFNA5* has been associated with severe combined immunodeficiency with sensitivity to ionizing radiation (SCID) disease [34]. *EFNA5* was implicated in repairing the DNA damage induced by ionizing radiation, and *FBXL17* is involved in U2OS osteosarcoma cellular sensitivity to ionizing radiation [35]. Finally, *FER* was also involved in activating of *ATM* protein as a central regulator of DNA damage response after RT. Finally, multi-gene expression visualization showed a high co-expression of *EFNA5* and *FBXL17* & *FER* in the minor salivary gland, suggesting these genes may play a role in the production and secretion of saliva. In summary, carriership of rs35542*A allele may indicate a dis-regulation of the expression of *EFNA5*, *FBXL17,* and *FER* genes. The dis-regulation, in turn, alters the cell response to enhanced production of reactive oxygen species induced by RT in the minor salivary gland leading eventually to cellular damage and a decrease in the production of saliva, the hallmark of xerostomia. Further research in transcriptome and proteome levels is needed to validate this hypothesis. More details of functional analysis are included in the Additional file 1: Supplementary Results.

Despite convincing functional analysis which supports the genetic finding in discovery study, we were not able to replicate our top hits. In general, non-replication is common in observational studies, mostly attributed to lack of sufficient study power. The non-replication may be explained by differences in the RT technologies used in the discovery and replication cohorts. The discovery cohort was treated with photon-based techniques, including intensity-modulated radiotherapy (IMRT) or Volumetric Arc Therapy (VMAT). Since 2018, intensity-modulated proton therapy (IMPT) has been clinically introduced in our centre, and patients were either treated with VMAT or IMPT based on predefined selection criteria [36]. Although the prescribed dose for tumour cells remained the same, the exposed dose to the relevant organs-at-risk and the spatial dose distributions per organ were significantly lower with IMPT, this may explain that the results from the discovery study could not be replicated. This is nicely illustrated by the fact that compared to the replication cohort, the discovery cohort showed significantly higher rates of acute RITs. Finally, the replication cohort included a limited number of patients with a lower incidence of RITs, which reduced the power of the replication study. Furthermore, the type of RT is likely to be a modifier, as it may change the incidence of RITs; however, this effect is independent of the carriership of genetic variants. Therefore change in the incidence of radiotoxicity due to the type of RT is deemed independent of genetic variation. Therefore, the difference in RT may not significantly modify the association between genetic variants and the studied acute RITs. Future studies with larger sample sizes are needed to determine whether the suggestive SNPs with marginally significant P-values are indeed associated with RITs in HNC patients.

In addition to patient-rated acute xerostomia, we studied seven other outcomes in which we did not find GWS associations, except for several suggestive associations. The lack of finding of GWS to outcomes is common. Previous GWASs have already observed the same phenomena [16, 30]. For example, Schack et al. studied nine HNC outcomes and found a single association with acute mucositis (Schack LMH et al. under review) but found no association for the rest of the eight studied outcomes. The differences in the frequency of the endpoints, patient selection, study setting, and treatment modalities reduce study power of GWAS for radiotoxicity.

This study has several strengths. HNC patients were selected out of a well-characterize prospective cohort, which is treated according to predefined guidelines over several years; by which radiation dosage was carefully assessed, the patient’s clinical response and side effect were systematically collected. The extent of acute RIT was characterized using AUC to estimate the average load of toxicity during the RT treatment period. We accounted for the patient- and treatment-related factors that influence the risk of acute RITs. Multiple imputation approach was used to handle missing values of RITs with high accuracy. This study also has some limitations. First, our modest sample size and subsequently low study power to detect SNPs with small effects. This study had 30% power to gain the GWS effect of (the rs2682585) SNP with a minor allele frequency (MAF) of 0.4 and an effect size of 1.4 on xerostomia. Second, functional analyses were done using online sources of expression data in a healthy population, as no patient data were available.

The clinical impact of identifying the genetic markers associated with RIT is yet to be defined. One immediate impact is including genetic variants in forming a prediction model that explains patients’ sensitivity, preceding RT. The ultimate question is whether the performance of currently used NTCP-models containing both dose and clinical parameters could be improved by addition of SNPs profiles for RIT.

## Conclusions

We identified a locus on 5q21.3 reaching GWS for association with radiation-induced acute xerostomia in the discovery study; however, we failed to replicate this finding in the replication study, likely due to the complexity of genetic studies in acute RITs, and subsequently the lack of sufficient study power. Nevertheless, in-silico functional analysis showed the region includes several ccREs likely to be involved in co-expression of the EFNA5, FBXL17, and FER genes in minor salivary glands. Therefore, future multicenter larger genetic studies are needed to verify our findings. In vitro/vivo functional analyses may reveal whether EFNA5-FBXL17-FER complex is causally associated with radiation-induced tissue damage in minor salivary glands and hence in the xerostomia pathogenesis.

## Supplementary Information

**Additional file 1: Introduction. Material and methods. Results. Table S1:** Endpoints. **Table S2:** Missing values in acute RITs. **Table S3.a, b & c:** Pre-imputation quality control genotyped samples. **Table S4:** Post-imputation quality control. **Table S5:** Heatmap of association of the predictors with acute RITs. **Figure S1:** PCA analysis for Ethnicity. **Figure S2:** Inclusion/exclusion criteria of study patients. **Figure S3:** Manhattan and QQ plots of GWA results for acute RITs. **Figure S4:** LD pattern of the 16 GWAs SNPs with patient-rated acute xerostomia. **References**.

**Additional file 2: Table S6:** Genome wide significant SNPs asscoiated with patient-rated acute xerostomia. **Table S7:** Suggestive SNPs associated with patient-rated acute xerostomia. **Table S8:** Suggestive SNPs associated with physician-rated acute xerostomia. **Table S9:** Suggestive SNPs associated with physician-rated acute dysphagia. **Table S10:** Suggestive SNPs associated with patient-rated acute sticky saliva. **Table S11:** Suggestive SNPs associated with physician-rated acute sticky saliva. **Table S12:** Suggestive SNPs associated with physician-rated acute mucositis. **Table S13:** Suggestive SNPs associated with STATphysician. **Table S14:** Suggestive SNPs associated with STATpatient

## Data Availability

The datasets used and analysed during the current study are available from the corresponding author on reasonable request.

## Abbreviations

ATM: Ataxia telangiectasia mutated
AUC: Area under the curve
ccRE: Cis-regulatory elements
DSB: Double-strand break
GW: Genome-wide
GWAS: Genome-wide association study
GWS: Genome-wide significant
HNC: Head and neck cancer
IMPT: Intensity-modulated proton therapy
IMRT: Intensity-modulated radiotherapy
LD: Linkage disequilibrium
MAF: Minor allele frequency
MI: Multiple imputation
NTCP: Normal tissue complication probability
QC: Quality control
RIT: Radiation-induced toxicity
RT: Radiotherapy
SCID: Sensitivity to ionizing radiation
SNPs: Single nucleotide polymorphisms
STAT: Standardized total average toxicity
VMAT: Volumetric arc therapy
