# Supplementary material for: A two-stage genome-wide association study of radiation-induced acute toxicity in head and neck cancer
Source: J Transl Med. 2021 Nov 27;19:481. doi: 10.1186/s12967-021-03145-1 (PMC8626989; doi:10.1186/s12967-021-03145-1)
Supplement: Supplementary file 1 — Additional file 1: Introduction. Material and methods. Results. Table S1: Endpoints. Table S2: Missing values in acute RITs. Table S3.a, b & c: Pre-imputation quality control genotyped samples. Table S4: Post-imputation quality control. Table S5: Heatmap of association of the predictors with acute RITs. Figure S1: PCA analysis for Ethnicity. Figure S2: Inclusion/exclusion criteria of study patients. Figure S3: Manhattan and QQ plots of GWA results for acute RITs. Figure S4: LD pattern of the 16 GWAs SNPs with patient-rated acute xerostomia. References. [file 12967_2021_3145_MOESM1_ESM.docx]

**Supplement to:**

A two-stage genome-wide association study of radiation-induced acute toxicity in head and neck cancer

*Elnaz Naderi. et al.*

**Table of contents**

**Introduction** 3

**Material and methods** 3

Eligibility criteria of participants 3

Assessment of RITs 3

Multiple imputations of missing value for toxicity 3

Genotyping, quality control, and imputation 4

Outcome modelling 5

**Results** 6

Multiple imputations (MI), AUC & STAT score 6

Clinical factors 6

Radiotoxicity endpoints 6

Patient-rated acute xerostomia 6

Physician-rated acute xerostomia 6

Physician-rated acute dysphagia 6

Patient-rated acute sticky saliva 7

Physician-rated acute sticky saliva 7

Physician-rated acute mucositis 7

Overall acute RIT: STAT_physician &_ STAT_patient_ 7

Combined study 8

*In-silico* functional analysis 8

**Supplementary Table S1**

Endpoints 10

**Supplementary Table S2**

Missing values in acute RITs 11

**Supplementary Table S3.a, b & c**

Pre-imputation quality control genotyped samples 12

**Supplementary Table S4**

Post-imputation quality control 15

**Supplementary Table S5**

Heatmap of association of the predictors with acute RITs 16

**Supplementary Figure S1**

PCA analysis for Ethnicity 17

**Supplementary Figure S2**

Inclusion/exclusion criteria of study patients 18

**Supplementary Figure S3**

Manhattan and QQ plots of GWA results for acute RITs 19

**Supplementary Figure S4**

LD pattern of the 16 GWAs SNPs with patient-rated acute xerostomia 25

**References** 26

**Introduction**

This supplementary appendix contains extended information on the material and methods and results sections as described in the main article. We supplied a separate supplementary table in Excel file format to present GWAS results for each of the acute radiation-induced toxicity (RIT) for single nucleotide polymorphism (SNP) with a suggestive association at a P-value<1.0x10^-5^_)_.

**Material and methods**

**Eligibility criteria of participants.** Eligibility criteria included histologically proven primary HNC, originating in the oral cavity, oropharynx, hypopharynx, nasopharynx, paranasal sinuses, and/or salivary glands; being treated with curative intent with definitive or postoperative radiotherapy (RT) either or not combined with systemic treatment; no history of prior radiation (in the head and neck area); having a northern European ethnicity; willing and able to comply with the study prescriptions, aged ≥18 years, signing informed consent, and a good level of understanding of the Dutch language. Patients who did not meet the inclusion criteria were excluded.

**Assessment of RITs.** Physician-rated acute toxicities including mucositis, dysphagia, xerostomia, and sticky saliva were registered according to the Common Toxicity Criteria of Adverse Events (version 4.0)(1). Patient-rated HNC symptoms were assessed using the EORTC QLQ-H&N35 questionnaire in addition to the EORTC QLQ-C30(2) (Supplementary Table S1). Both patient- and physician-rated scores were available for xerostomia and sticky saliva. However, only physician-rated gradings were available for dysphagia and mucositis. The definition, scoring, and availability of acute (during RT; week one to seven) RITs are listed in Supplementary Table S1.

**Multiple imputations of missing value for toxicity.** Missingness in measurements of outcomes or predictors is a potential source of bias. Missing data is occurring especially in patients’ data collected by their treating radiation oncologists during routine clinical practice. Missing data must be appropriately handled in the statistical analysis. We observed varying percentage of missingness across toxicity endpoints (Supplementary Table S2). To impute missing values of toxicity endpoints, we applied Multiple Imputation (MI) as implemented in the MICE package of the analytical interface R. Missing values were imputed using a series of univariate conditional imputation models. To ensure that the imputation model preserved the relationships between the variables of interest, we formed an imputation model that contained two levels of predictive variables. First, the clinically and biologically relevant variables which would have been included in the final analysis model (given the study’s main research question) and secondly a large set of explanatory (auxiliary) variables that would have not been included in the final analysis model but were highly correlated (i.e. correlation coefficient of >0.90) with the toxicity assessments. Linear regression (for continuous outcomes), logistic regression (for binary outcomes), and multinomial logit model (for outcomes with more than two categories) methods were used to form the imputation model. We repeated the MI approach 100 times to reduce the uncertainty in predicting the missing values, which yielded 100 completed datasets compromised of imputed and observed values. We calculated 100 AUC scores per each of the six acute toxicity endpoints. Next, the mean of the 100 AUCs was estimated per each acute toxicity endpoint.

**Genotyping, quality control, and imputation of non-genotyped variants.** Among 1,429 patients, germline DNA of 607 patients was genotyped on Illumina human Core (Infinium OmniExpress-24 ver 1.3 kit with one million tag SNPs) and for 822 subjects (495 of discovery samples and 327 replication samples) on Illumina global screening array (GSAMD_24V1&V3 with 700,000 tag SNPs). Quality control (QC) procedures were performed using standard procedures. First, SNPs and samples with call rates below 98% were removed. Next, a strict SNP QC only for subsequent sample quality control steps was conducted, including i.) a minor allele frequency (MAF) threshold of >10%, and followed by linkage disequilibrium (LD) based SNP pruning at linkage index of (R^2^) <0.2, we assessed gender mismatch using the actual X chromosome homozygosity index (F) of >0.8 representing for the male gender, and an F of <0.2 represents a female gender. Relatedness was evaluated by pairwise identity by descent (IBD) values when duplicate samples were considered by a pihat>0.8 who were removed, and remaining pairs were manually checked. After removing QC failed samples, we performed a regular SNP QC conditioned at an SNP call rate of >98%, and MAF>1%. We checked the ethnicity of subjects using multidimensional scaling (MDS) clustering of our samples with Hapmap Phase 3 individuals using EIGENSTRAT. Samples that deviated more than 3 SD from the mean of their closest clusters were removed (Supplementary Figure S1). Furthermore, individuals were assigned to populations based on principal component analysis (PCA). PCA was performed using EIGENSTRAT(3). The top 10 PCA eigenvectors were included in the final models for all endpoints. Next, strand ambiguous SNPs and duplicate SNPs were removed (Supplementary Table S3a,b&c). Finally, SNPs were imputed on the Michigan server using the HRC r1.1 2016 reference panel with European samples after phasing with Eale v2.3. We performed post-imputation QC involved removing SNPs with an imputation quality (info) score of R^2^<0.3, or with a MAF of <0.01, and SNPs that had a discordant MAF (maximum allowed difference<0.15) compared to the reference panel, or strand ambiguity AT/CG SNPs, or multi-allelic SNPs (Supplementary Table S4).

**Outcome modelling.** We assessed the endpoints using two different scoring systems. First, since acute RIT generally increases during treatment, we applied the concept of the area under the curve (AUC) to generate an overall measure of acute toxicity during RT treatment up to seven weeks after RT per individual patient using the following equation:


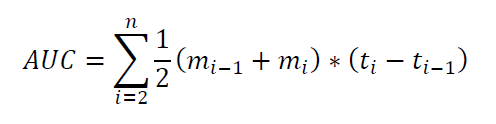


Where m_i_ shows the toxicity score at a specific time point (*i*) and t_i_ shows the time interval. Calculation of AUC per each RIT was applied to each of the imputed and observed datasets, which yielded 100 AUC scores per each RIT. By taking the average of the 100 AUCs, we estimated a unique AUC score for each of the acute RIT.

Second, to achieve a composite score representing the general acute RIT, we used the standardized total average toxicity (STAT) method. STAT acute score was calculated as follows(4):

Z_k,i_ = (s_k,I_ - Mean_i_) / Standard Deviation_i_

where “s” is the AUC score for a specific endpoint (i), in each individual (k). Mean_i_ and Standard Deviation_i_ are taken over all cases. The STAT score for patient k, STAT_k_, is simply the average of all z scores for that patient:

STAT_k_ = mean Z_k,i_

STAT*_physician_* and STAT*_patient_* included all the acute toxicities which have been reported by physician and patient, respectively. There is a difference in the grading of physician-rated toxicity endpoint graded as 0, 1, 2, 3, or 4, and patient-rated symptom scores graded as none, slight, moderate, severe.

**Association analyses.** Multivariate linear regression was used to estimate the association of the additive effect of SNPs effect allele with AUCs and STAT scores. Multivariable models were adjusted for the aforementioned covariates and the top 10 PCA eigenvectors. SNPs were included as the number or dosage of effect alleles in (imputed) genotypes resulting in a regression coefficient for one copy increase in effect allele. A genome-wide P-value<5.0x10-8 was considered statistically genome-wide significant (GWS), and a P-value<1.0x10-5 was considered a suggestive association. PLINK/1.90b3.44(5) and R statistical package v3.3.0 were used for data analysis. The results were visualized with Manhattan and Q-Q plots using qqman package(6).

**Results**

**Multiple imputations (MI), AUC & STAT score.** A mean of AUCs represents an average of toxicity per week during the RT plan. Table 2 (main text) describes the distribution of acute RITs in the discovery and replication study. In the combined study, the means for physician-rated acute endpoints were 1.91 (SD ±0.51) for xerostomia, 1.85 (±0.81) for dysphagia, 1.94 (±0.72) for mucositis, and 1.70 (±0.45), for sticky saliva with higher values indicating more toxicity. The mean of AUC for patient-rated acute endpoints were 2.21 (±0.73) for xerostomia and 2.10 (±0.73) for sticky saliva (Figure 1, main text). STAT*_physician_* score was calculated using the AUC of xerostomia, dysphagia, mucositis, and sticky saliva for physician-rated acute RITs. The mean (SD) of STAT*_physician_* score was zero (±0.78). STAT*_patient_* score for patient-rated acute RITs was calculated using AUC of xerostomia and sticky saliva with a mean zero (±0.90).

**Clinical factors.** Supplementary Table S5 presents the details of association for all predictors with each of the acute RITs in the discovery study. Two predictors, including baseline toxicity and volume surrogate, highly correlated with all acute RITs. The extent of association between baseline toxicity and acute endpoints varied from beta=0.36 to 0.70. Volume surrogate was associated with acute RITs by effect (beta) range of 0.35 to 0.76.

**GWASs of acute RITs**

**Patient-rated acute xerostomia.** This is explained in the main part.

**Physician-rated acute xerostomia.** In the discovery study, overall 42 suggestive SNPs spread across 9 loci showed associations with physician-rated acute xerostomia of which the top SNP was rs17716780 with per-risk allele effect size of 0.12 (0.07 to 0.16; P-value<=1.09x10^-7^). None of these suggestive SNPs were replicated at the statistical level (Supplementary Table S8).

**Physician-rated acute dysphagia.** There were 78 SNPs in 18 loci that were identified as suggestively associated with physician-rated acute dysphasia in the discovery study. The top SNP was rs73614646 with an effect of 0.32 (0.20 to 0.45; P-value<=3.71x10^-7^) per one increase of risk G allele. In the replication study, the73 available (out of 78 suggestive SNPs) were included in the analysis (Supplementary Table S9), of which none were replicated. The meta-effect size for the rs73614646*G allele was 0.27 (95%CI 0.06 to 0.16), which failed to reach the statistical GWS threshold (P*_meta_* = 2.21x10^−6^).

**Patient-rated acute sticky saliva.** Thirty-eight SNPs across 17 genomic regions, showed suggestive associations. The top SNP was rs6987582 with an effect size of -0.16 (-0.22 to -0.09) for the risk C allele with a P-value of <=7.10x10^-7^. In the replication study, the 36 available suggestive SNPs were analysed (Supplementary Table S10), of whom none neared a GWS association in the meta-analysis.

**Physician-rated acute sticky saliva.** We observed 62 SNPs distributed in 10 genomic regions. The top SNP was rs1535557 with an effect size of 0.11 (0.06 to 0.15; P-value <=7.47x10^-7^) per one copy increase in the risk G allele. The replication study showed none of the suggestive SNPs was significantly associated with the outcome, either by replication or overall meta-analysis (Supplementary Table S11). The meta-effect size for the rs1535557*G allele was 0.09 (95%CI 0.05 to 0.13) at P*_meta_* = 2.14x10^−6^, which was less than GW significant.

**Physician-rated acute mucositis.** The analysis of the discovery cohort found 65 suggestive SNPs mapped in 15 genomic regions. Here, the top SNP was rs75271259 which showed an effect size of -0.33 (-0.46 to -0.21; P-value<=6.29x10^-7^) per one copy increase in the effect A allele. Again, the replication study of 63 (out of 65 suggestive SNPs) was not able to replicate any of these SNPs at a statistically significant level, nor did the meta-analysis (Supplementary Table S12). The meta-effect size, of the rs75271259*A allele, was -0.28 (95%CI -0.39 to -0.16) at P*_meta_* = 3.34x10^−6^, which did not reach a GWS level.

**Overall acute RIT.** To gain more power, we combined individual RIT into a composite score that represents overall acute RIT, namely STAT*_physician_* and STAT*_patient_*.

**STAT*_physician_*.** We found 26 suggestive SNPs distributed across 13 genomic regions associated with STAT*_physician_*. The rs117680507 was the most significantly associated SNP with an effect size of -0.65 (-0.91 to -0.40; P-value<=5.25x10^-7^) per one copy of the risk C allele. In the replication study, we found no statistically significantly associated SNP in the replication cohort or the overall meta-analysis (Supplementary Table S13). The most significant associated SNP in the meta-analysis was the rs116340739*C with a meta-effect size of 0.46 (95%CI 0.26 to 0.65; P*_meta_* = 4.64x10^−6^).

**STAT*_patient_*.** A total of 74 suggestive SNPs across 20 genomic regions were suggestively associated with STAT*_patient_* in discovery analysis, rs9521025 being the top associated SNP with an effect size of 0.22 (0.14 to 0.31; P-value<=3.04x10^-7^) per increase C allele. In the replication study, none of the suggestive SNPs from the discovery study was statistically associated with STAT*_patient_* (Supplementary Table S14). The meta-effect size for rs9521025*C allele was 0.20 (95%CI 0.13 to 0.27; P*_meta_* = 2.24x10^−7^).

**Combined GWAS study.** The additive effect of more than six million SNPs was analysed for their associations with acute toxicity endpoints in the combined discovery and replication sets consisting of 1,279 HNC patients. Given we used different Illumina array chips to genotype the patients, an additional proxy co-variable was included in the model to correct for possible batch effects. None of the tested SNPs was significantly associated with any of the eight tested acute RITs. There were 710 suggestive SNPs at P-value<1.0x10^-5^ located in 137 genomic regions associated with all endpoints. Supplementary Figure S3 presents Manhattan and Q-Q plots of combined GWA analyses across all endpoints.

***In-silico* functional analysis**

Given the limited statistical power of the replication cohort, we decide to perform *in-silico* sequencing and functional analysis on the identified genome-wide associated SNPs in the discovery study (GWS*_discovery_* SNPs). Annotation analysis showed the GWS*_discovery_* SNPs associated with patient-rated acute xerostomia were located in a noncoding genomic-block on chr5 from 107085963 to107110731 base pair on genome build GRCh37.p13.chr5. The genome sequence of this block is known to be a regulatory region, possibly homing some cis-Regulatory Elements (ccREs). Using ENCODE functional data, we found this genomic block constitutes several ccREs. Furthermore, these ccREs were subjected to epigenetic activity by histone modification of the H3K27Ac (acylation of lysine 27 of histone 3) and H3K4me3 (methylation of lysine 4 of histone 3). Interestingly, H3K4me3 is implicated in the non-homologous end-joining pathway which promotes the repair of double-strand DNA breaks (DSBs)(7). DSBs that are induced by ionizing radiation during RT, are considered as the most lethal form of DNA damage and a primary cause of cell death(8). Exploring the genome map showed Ephrin-A5 (*EFNA5),* F-Box and Leucine Rich Repeat Protein 17 (*FBXL17),* and FER Tyrosine Kinase (*FER)* genes are the most likely genes related to ccREs on which the GWS_dis_ SNPs reside (Figure 2C, main text). The next question was if the activities of the neighbouring genes are related to radiosensitivity*.* Using GeneCards and MalaCards, we found that *EFNA5* has been previously associated with severe combined immunodeficiency with sensitivity to ionizing radiation (SCID) disease. The common characteristic of all types of SCID is the absence of T-cell-mediated cellular immunity due to a defect in T-cell development, due to increased radiosensitivity, known as radiosensitivity-SCID.(9) Moreover, a previous study showed *EFNA5* is involved in repairing the DNA damage induced by ionizing radiation. The second gene was, *FBXL17*. A study aimed to identify novel DNA damage response genes using a genome-wide loss-of-function screen in U2OS osteosarcoma cells showed *FBXL17* depletion led to cellular sensitivity to ionizing radiation(10). As for *FER*, knock-down of Fer tyrosine kinase (*FER)*, impaired cell-cycle progression, imposed programmed cell death in colon carcinoma cells (CCD-33Co), and increased the level of reactive oxygen species(11). A down-regulation of *FER* led to the activation of the ataxia telangiectasia mutated (*ATM*) protein and its down-stream effector-p53. The role of *ATM* in radio-sensitivity, as central regulators of DNA damage response after RT, is endorsed by previous findings on its significant association with RITs in breast and prostate cancers(12). Follow-up analysis of the GTEx tissue-specific expression profiles showed the nearest gene, *EFNA5*, is highly expressed in minor salivary glands and also in the skin, and pituitary glands (Figure 3A, main text). For the *EFNA5*, the maximum expression with a median of 20.175 TPM belongs to skin-sun exposed tissues and the minimum expression belongs to whole blood with a median of 0.084 TPM. Among all tissues, the minor salivary gland has the fourth-ranking of high expression with a median expression level equal to 14.04 TPM. Using multi-gene query visualization in GTEx showed these three genes have the same co-expression pattern in secretory tissues including the minor salivary gland, vagina, and pituitary and also in skin-sun/non-sun exposed (Figure 3B, main text). This evidence suggested that these genes may a play role in the production and secretion of extracellular fluid in the human body, including saliva.

**Supplementary Table S1.**

Definition, scoring and availability of all acute outcomes.

| **End points** | **Definition** | **Type of Report** | **Scoring** |
| --- | --- | --- | --- |
| **Mucositis** | Mucositis is an acute radiation effect that takes place in mucosal membranes. It is characterized by loss of the epithelial surface resulting in a superficial fibrin covered defect in the mucosal membrane. | Physician | 0= no mucositis  1= erythema  2= patchy mucositis  3= confluent mucositis  4= deep ulceration |
| **Dysphagia** | Dysphagia is characterized by having difficulties in swallowing food. It is often graded according to the extent of the problem. | Physician | 0= regular diet  1= soft food  2= liquids only  3= tube feeding dependent |
| **Xerostomia** | Xerostomia is characterized by dryness of the mouth due to a reduced production of saliva. | Physician  and  patient | 0= Grade 0  1= mild xerostomia, no altered diet  2=moderate xerostomia, altered diet  3= complete dryness |
| **Sticky saliva** | Sticky saliva represents a different expression of salivary gland dysfunction. Whereas xerostomia is mainly related to the quantity of produced saliva, sticky saliva is closer related to the quality of the produced saliva. | Physician  and  patient | 0= no sticky saliva  1= mild sticky saliva, normal diet  2= moderate sticky saliva, altered diet  3= severe sticky saliva |

| **Supplementary Table S2.**  The number of subjects with (and percentage of) missingness of acute RITs among 1,279 HNC patients (%). | | | | | | | | | |  |
| --- | --- | --- | --- | --- | --- | --- | --- | --- | --- | --- |
| **Visiting week** | | | | | | | | | |  |
| **Physician rated** | | **Endpoints** | **week1** | **week2** | **week3** | **week4** | **week5** | **week6** | **week7** |  |
|  |  | Mucositis | 33(2.6) | 27(2.1) | 20(1.6) | 33(2.6) | 61(4.8) | 153(9.8) | 327(25.6) |  |
|  |  | Dysphagia | 38(3.0) | 30(2.4) | 20(1.6) | 36(2.8) | 60(4.7) | 153(9.8) | 325(25.4) |  |
|  |  | Xerostomia | 33(2.6) | 26(2.0) | 20(1.6) | 32(2.5) | 60(4.7) | 154(9.9) | 326(25.5) |  |
|  |  | Sticky saliva | 37(2.9) | 29(2.3) | 20(1.6) | 34(2.7) | 59(4.6) | 153(9.8) | 325(25.4) |  |
| **Patient rated** | | Xerostomia | 96(7.5) | 77(6.0) | 59(4.6) | 69(5.4) | 97(7.6) | 167(13.1) | 408(31.9) |  |
|  |  | Sticky saliva | 102(8.0) | 83(6.5) | 66(5.2) | 68(5.3) | 100(7.8) | 162(12.7) | 410(32.1) |  |

| **Supplementary Table S3a.**  Pre-imputation quality control (QC) process of genotypes for the first cohort as discovery cohort genotyped by Illumina human Core (Infinium OmniExpress-24 ver 1.3) array. | | | | |
| --- | --- | --- | --- | --- |
| **QC steps** | **SNP start** | **SNP**  **end** | **Subjects**  **Start** | **Subjects end** |
| DATA set 1 | | | | |
| Remove 33 samples without clinical data | 964,193 | 964,193 | 607 | 574 |
| Remove SNPs with missingness > 0.05 | 964,193 | 960,717 | 574 | 574 |
| Remove samples >2% missing genotypes | 960,717 | 960,717 | 574 | 572 |
|  |  |  |  |  |
| *Strict SNP QC -- remove SNPs with maf < 10 %* | *960,717* | *537,239* | *572* | *572* |
| *Prune SNPs on 0.2 max. LD* | *537,239* | *113,358* | *572* | *572* |
| *Check gender and heterozygous haploid genotypes* | *113,358* | *11,3285* | *572* | *568* |
| *Check heterozygosity* | *113,285* | *113,285* | *568* | *568* |
| *Check relatedness (identical – pi_hat > 0.8)* | *113,285* | *113,285* | 568 | 566 |
| Remove all bad samples strict QC (6 sample) | 960,717 | 960,717 | 572 | 566 |
| General QC : | | | | |
| –Exclude SNPs with missingness > SNPs 2% | 960,717 | 953,863 | 566 | 566 |
| –Exclude SNPS with MAF < 1% | 953,863 | 669,897 | 566 | 566 |
| Exclude Ethnicity outliers based on MDS plot HapMap | 669,897 | 669,897 | 566 | 558 |
| Re-Run QC steps | | | | |
| Remove 14 samples based on strict QC above | 964,193 | 964,193 | 574 | 560 |
| Remove SNPs with missingness > 0.05 | 964,193 | 960,685 | 560 | 560 |
| Remove subjects >2% missing genotypes | 960,685 | 960,685 | 560 | 558 |
| SNP QC – remove SNPS with missing SNPs > 2%, | 960,685 | 954,022 | 558 | 558 |
| SNP QC – Remove SNPS with MAF < 1% | 954,022 | 668,671 | 558 | 558 |
| Remove SNPs with heterozygous. haploid genotypes | 668,671 | 668,405 | 558 | 558 |
| Separate autosomal and X chromosomes  X chromosome  chr 1-22 | 668,405 | 14,730  652,397 | 558  558 | 558  558 |
| Coordinate SNPs with HRC/1000G for strand, id names, positions, alleles, ref/alt assignment  X chromosome  chr 1-22 | 14,730  652,397 | 14,226  627,697 | 558  558 | 558  558 |

| **Supplementary Table S3b.**  Pre-imputation quality control (QC) process of genotypes for the second cohort as discovery cohort genotyped by illumine Global Screening Array ( GSAMD_24V1). | | | | |
| --- | --- | --- | --- | --- |
| **QC steps** | **SNP**  **start** | **SNP**  **end** | **Subjects**  **Start** | **Subjects end** |
| Data set 2 | | | | |
| Remove 82 samples without clinical data | 692,367 | 692,367 | 495 | 413 |
| Remove SNPs with missingness > 0.05 | 692,367 | 688,626 | 413 | 413 |
| Remove samples >2% missing genotypes | 688,626 | 688,626 | 409 | 409 |
| *Strict SNP QC -- remove SNPs with maf < 10 %* | 688,626 | *239,148* | *409* | *409* |
| *Prune SNPs on 0.2 max. LD* | 688,626 | *88,725* | *409* | *409* |
| *Check gender and heterozygous haploid genotypes* | *88,725* | *88,412* | *409* | *409* |
| *Check heterozygosity* | *88,412* | *88,412* | *409* | *409* |
| *Check relatedness (identical – pi_hat > 0.8)* | *88,412* | *88,412* | 409 | 408 |
| Remove all bad samples strict QC (1 sample)  General QC : | 688,626 | 688,626 | 409 | 408 |
| –exclude SNPs with missingness > SNPs 2% | 688,626 | 680,041 | 408 | 408 |
| –Exclude SNPS with MAF < 1% | 680,041 | 489,270 | 408 | 408 |
| Exclude Ethnicity outliers based on MDS plot HapMap | 489,270 | 489,270 | 408 | 399 |
| **Re-Run QC steps** | | | | |
| Remove 10 samples based on strict QC above | 692,367 | 692,367 | 413 | 403 |
| Remove SNPs with missingness > 0.05 | 692,367 | 688,735 | 403 | 403 |
| Remove samples >2% missing genotypes | 688,735 | 688,735 | 403 | 399 |
| SNP QC - missing SNPs 2%, | 688,735 | 678,316 | 399 | 399 |
| SNP QC - MAF 1% | 678,316 | 491,988 | 399 | 399 |
| Remove het. haploid genotypes | 491,988 | 490,874 | 399 | 399 |
| Separate autosomal and X chromosomes  X chromosome  chr 1-22 | 490,874 | 10,190  479,552 | 399  399 | 399  399 |
| Check against HRC/1000G for strand, id names, positions, alleles, ref/alt assignment  X chromosome  chr 1-22 | 10,190  479,552 | 10,005  467,258 | 399  399 | 399  399 |

| **Supplementary Table S3c**  Pre-imputation quality control (QC) process of genotypes for the third cohort as replication cohort genotyped by illumine Global Screening Array (GSAMD_24V3). | | | | |
| --- | --- | --- | --- | --- |
| **QC steps** | **SNP**  **start** | **SNP**  **end** | **Subjects**  **Start** | **Subjects end** |
| Data set 3 | | | | |
| Remove SNPs with missingness > 0.05 | 725,831 | 720,745 | 327 | 327 |
| Remove samples >2% missing genotypes | 720,745 | 720,745 | 327 | 326 |
| *Strict SNP QC -- remove SNPs with maf < 10 %* | 720,745 | *245,936* | *326* | *326* |
| *Prune SNPs on 0.2 max. LD* | *245,936* | *91,460* | *326* | *326* |
| *Check gender and heterozygous haploid genotypes* | *91,460* | *91,460* | *326* | *324* |
| *Check heterozygosity* | *91,460* | *91,354* | *324* | *324* |
| *Check relatedness (identical – pi_hat > 0.8)* | *91,354* | *91,354* | 324 | 324 |
| Remove all bad samples strict QC (3 sample)  General QC : | 720,745 | 720,745 | 327 | 324 |
| –exclude SNPs with missingness > SNPs 2% | 720,745 | 680,041 | 324 | 324 |
| –Exclude SNPS with MAF < 1% | 709,454 | 500,440 | 324 | 324 |
| Exclude Ethnicity outliers based on MDS plot HapMap | 500,440 | 500,440 | 324 | 322 |
| **Re-Run QC steps** | | | | |
| Remove 4 samples based on strict QC above | 725,831 | 725,831 | 327 | 323 |
| Remove SNPs with missingness > 0.05 | 725,831 | 720,628 | 323 | 323 |
| Remove samples >2% missing genotypes | 720,628 | 720628 | 323 | 322 |
| SNP QC - missing SNPs 2%, | 720,628 | 720,628 | 322 | 322 |
| SNP QC - MAF 1% | 709,625 | 499,472 | 322 | 322 |
| Remove het. haploid genotypes | 499,472 | 499,105 | 322 | 322 |
| Separate autosomal and X chromosomes  X chromosome  chr 1-22 | 499,105 | 19,812  479,293 | 322  322 | 322  322 |
| Check against HRC/1000G for strand, id names, positions, alleles, ref/alt assignment  X chromosome  chr 1-22 | 19,812  479,293 | 10,005  477,069 | 322  322 | 322  322 |

| **Supplementary Table S4**  Post-imputation quality control (QC) process by the study cohorts. | | | | | | | |  |
| --- | --- | --- | --- | --- | --- | --- | --- | --- |
| **Dataset** | **Genotyped SNPs** | **Imputed SNPS** | **Fail SNPs*** | **Excluded Strand ambiguous AT/CG** | **Total Excluded SNPs** | **QC passed SNPs** | **Merged SNPs** | **Total combined SNPs** |
| **First** | 627,697 | 38,500,782 | 31,304,800 | 1,157,016 | 32,461,816 | 6,665,906 | 6,334,277 | 6,663,871 |
| **Second** | 467,258 | 38,664,320 | 31,288,130 | 1,165,480 | 32,453,610 | 6,677,968 |  |  |
| **Third** | 477,069 | 38,452,840 | 30,644,468 | 1,124,489 | 31,888,957 | 6,563,883 |  |  |
| * Fail if any SNP had a minor allele frequency (MAF) <0.01, imputation quality of information (INFO) <0.3, compare MAF with HRC reference as maximum allowed difference between HRC reference panel and experiment <0.15) and had more than 2 alleles. | | | | | | | |  |

| **Supplementary Table S5**  Association (effect-size) the predictors with acute RT toxicities in discovery cohort from univariate analysis. | | | | | | | | | | |
| --- | --- | --- | --- | --- | --- | --- | --- | --- | --- | --- |
|  | | **Age** | **Gender** | **Tumour site** | **T-stage** | **N-stage** | **Chemo**  **therapy** | **Post**  **operative RT** | **Volume Surrogate** | **Baseline**  **toxicity** |
| Mucositis  (physician report) | -0.01 | 0.01 | -0.03 | -0.05 | -0.45 | 0.28 | -0.02 | 0.71 | 0.54 |  |
| Dysphagia  (physician report) | -0.03 | 0.07 | 0.02 | 0.13 | -0.12 | 0.35 | -0.05 | 0.34 | 0.70 |  |
| Xerostomia  (physician report) | 0.01 | 0.13 | 0.00 | -0.11 | -0.45 | 0.12 | -0.01 | 0.76 | 0.47 |  |
| Sticky saliva  (physician report) | -0.06 | 0.04 | 0.13 | 0.01 | -0.29 | 0.15 | -0.02 | 0.57 | 0.36 |  |
| Xerostomia  (patient report) | 0.07 | 0.18 | -0.01 | -0.09 | -0.43 | 0.05 | -0.16 | 0.66 | 0.58 |  |
| Sticky saliva  (patient report) | -0.02 | 0.02 | 0.11 | 0.02 | -0.21 | 0.09 | -0.13 | 0.49 | 0.61 |  |
| STAT physician | -0.03 | 0.11 | 0.04 | -0.01 | -0.42 | 0.29 | -0.06 | 0.76 | - |  |
| STAT patients | 0.03 | 0.11 | 0.06 | -0.04 | -0.36 | 0.08 | -0.17 | 0.65 | - |  |

The effect sizes and its significance present by number and cell colour (defined by the below legend) respectively.

| Not significant (p=value > 0.05) |  |
| --- | --- |
| Significant (p=value =< 0.05) |  |
| Significant (p=value < 0.01) |  |

**Principal component analysis (PCA)** was used to identify and exclude patients with non-European ancestry. We performed PCA by combining the genotype of the study cohorts, with those from the Europeans HapMap genotypes data (release 23) using EIGENSOFT software. Patients were excluded if the location of the sample on main eigenvectors 1 and 2 were more than 3 standard deviations from the mean of the location of the Europeans’ genotypes.


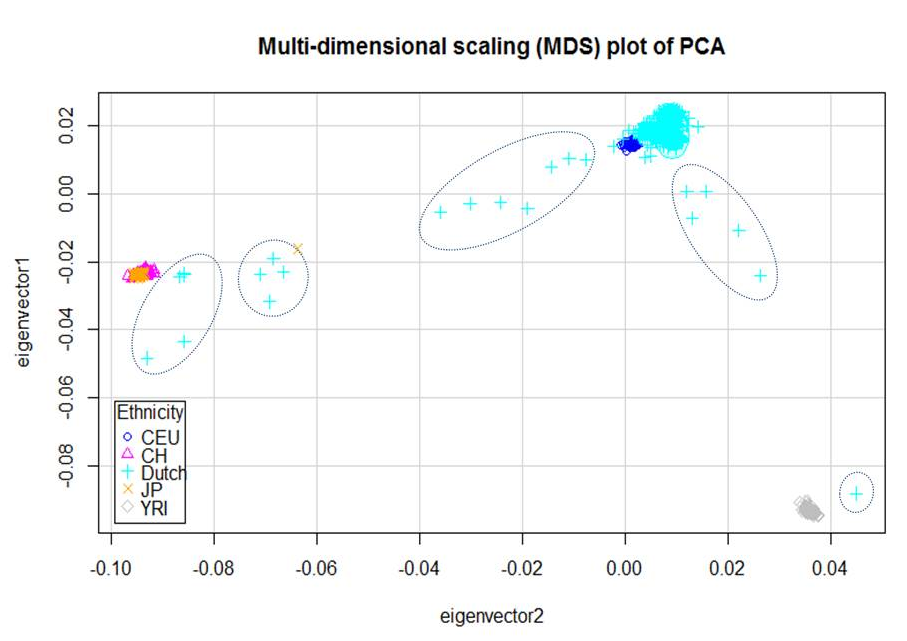


**Supplementary Figure S1:** Based on PCA analysis, 22 samples (enclosed among 5 clusters) deviated more than 3SD from the mean of CEU cluster who were identified as non-European ancestry in UMCG-HNC cohorts which were excluded for further analysis.


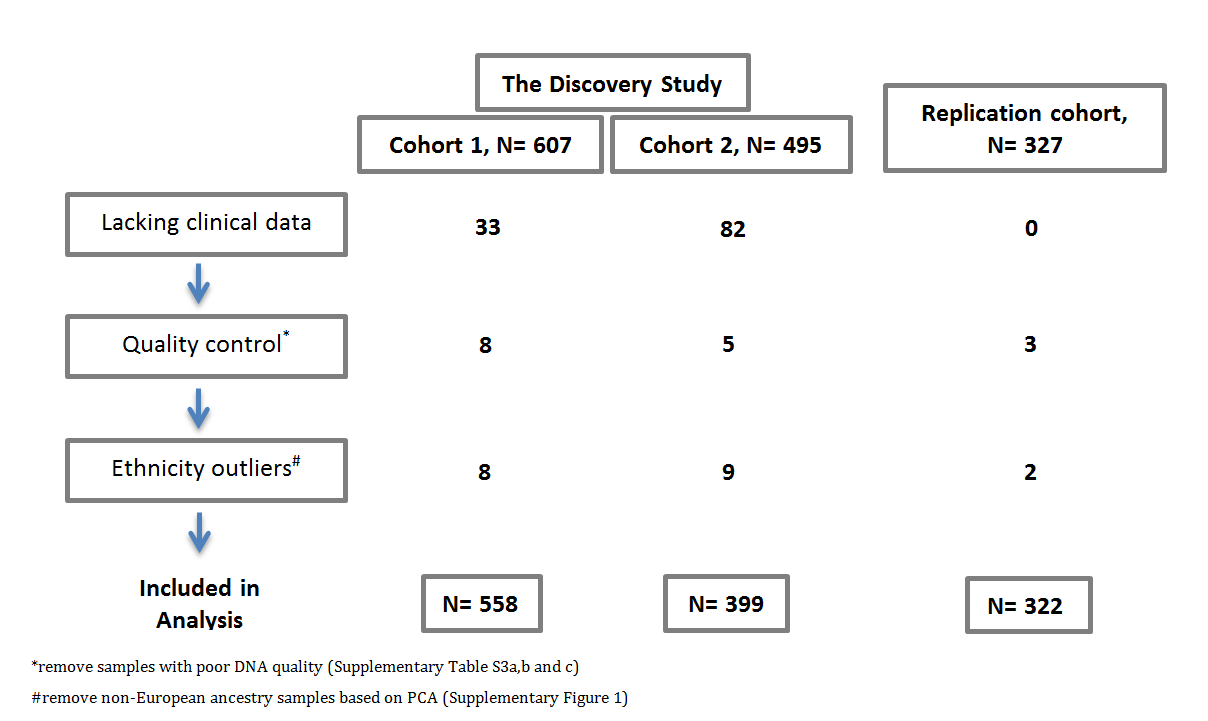


**Supplementary Figure S2.** Inclusion/exclusion of study subjects within the study cohorts. Figures represent the number of patients excluded.

**Supplementary Figure S3:** Manhattan (right) and QQ (left) plots of genome wide association analysis for the tested acute RITs in HNC patients. **Multi-tracks Manhattan plot**: both discovery and combined analysis in the same plot per each toxicity endpoint. The yellow and blue dots represent discovery and combined analysis respectively. The *X* axis shows location in the genome. The *Y* axis shows −log_10_ P-values for the association of each of the SNPs to the desired outcome. The black line shows the threshold for genome-wide (GW) significance (P-value<5×10^–8^) and light gray line shows the threshold for suggestive associations (P-value<1×10^–5^). At the bottom, SNP-density plot displays the distribution and density of the SNPs across all chromosomes. Colours separate windows with different SNP density from low to high by green to red colours respectively. **QQ plot of combined analysis**: The *Y* axis shows observed −log_10_ P-values, and the *X* axis shows the expected −log_10_ P-values. Each SNP is plotted as a dark blue dot, and the red line indicates null hypothesis of no true association. Deviation from the expected P-value distribution is evident only in the tail area, and along with the estimated lambda coefficients, suggesting that population stratification was adequately controlled.


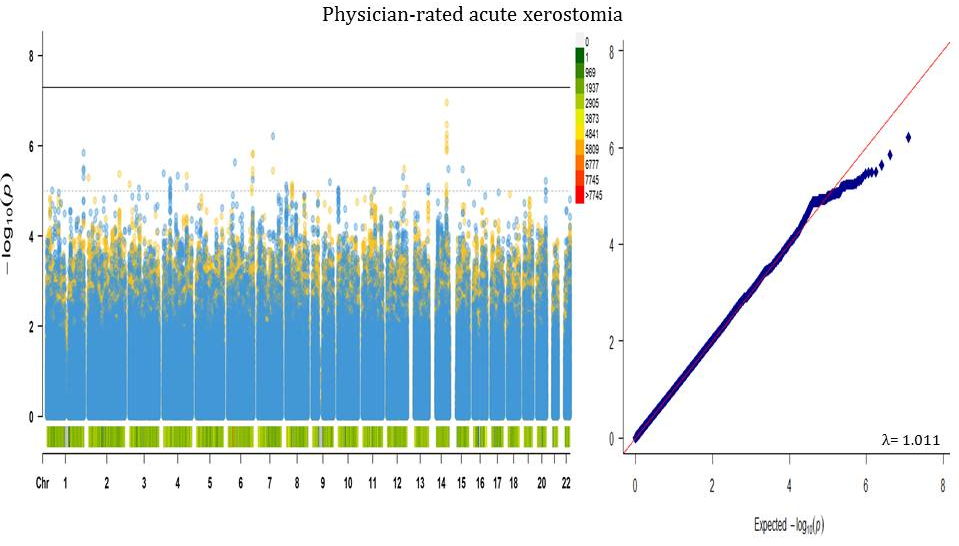


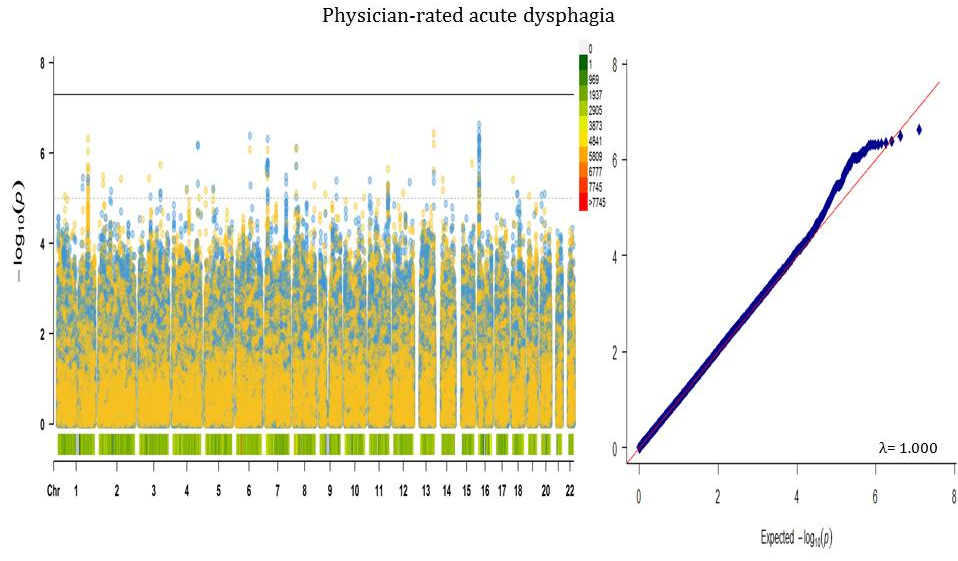


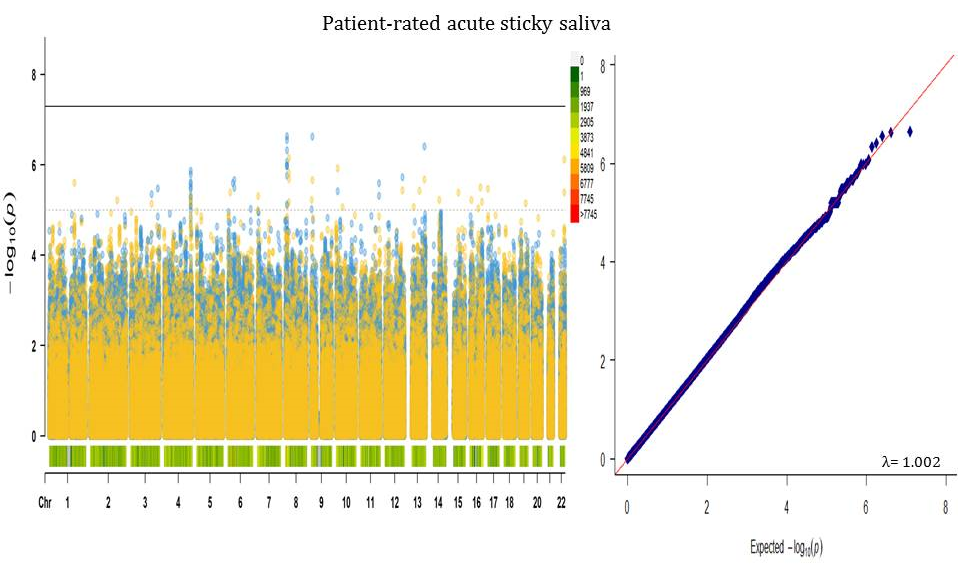


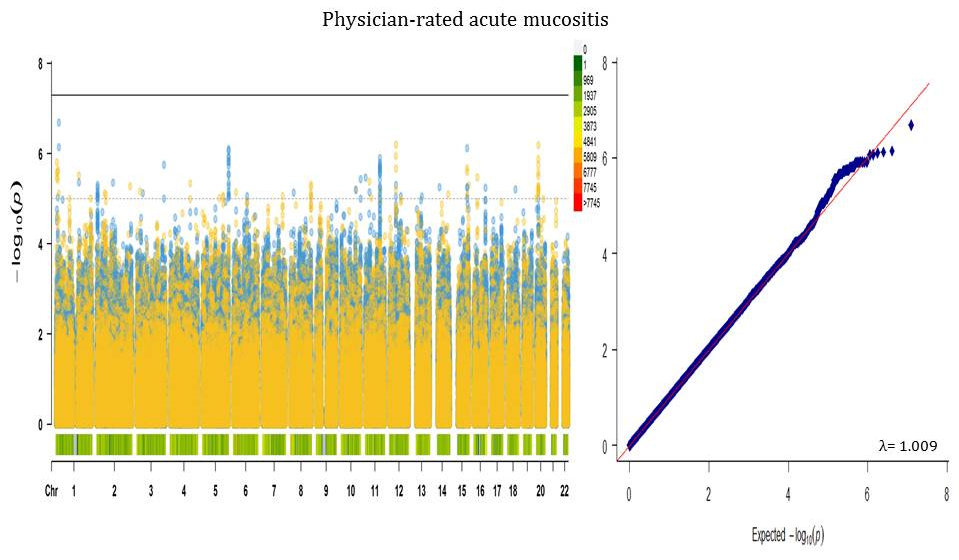


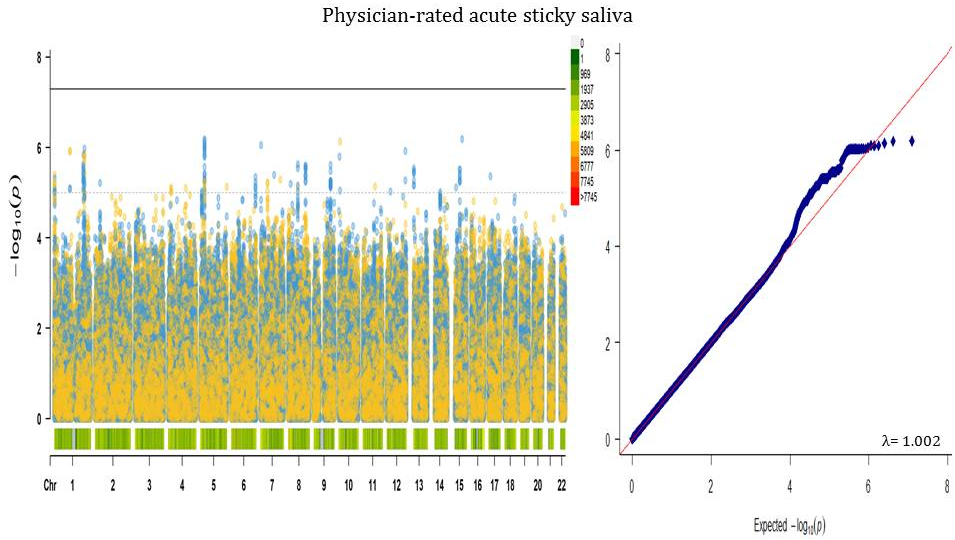


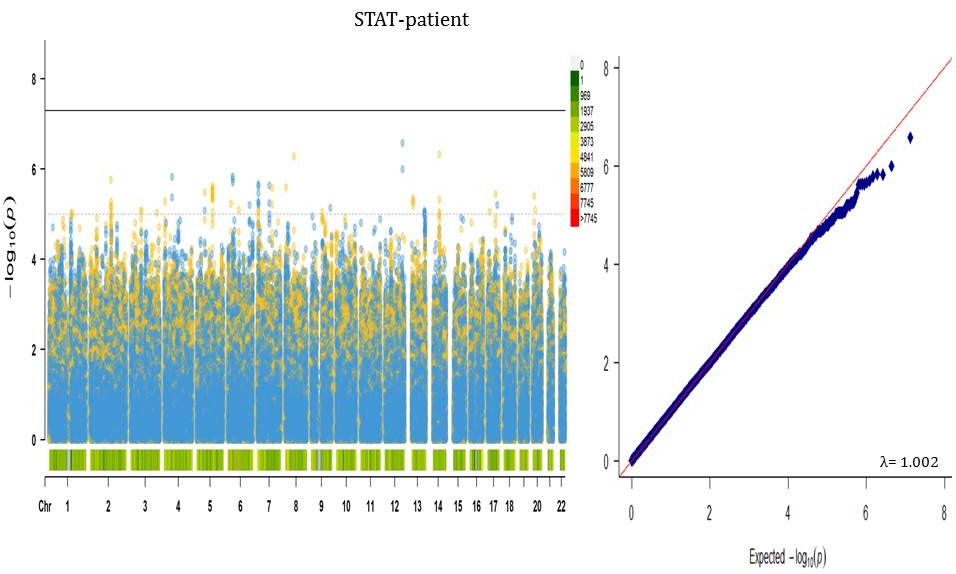

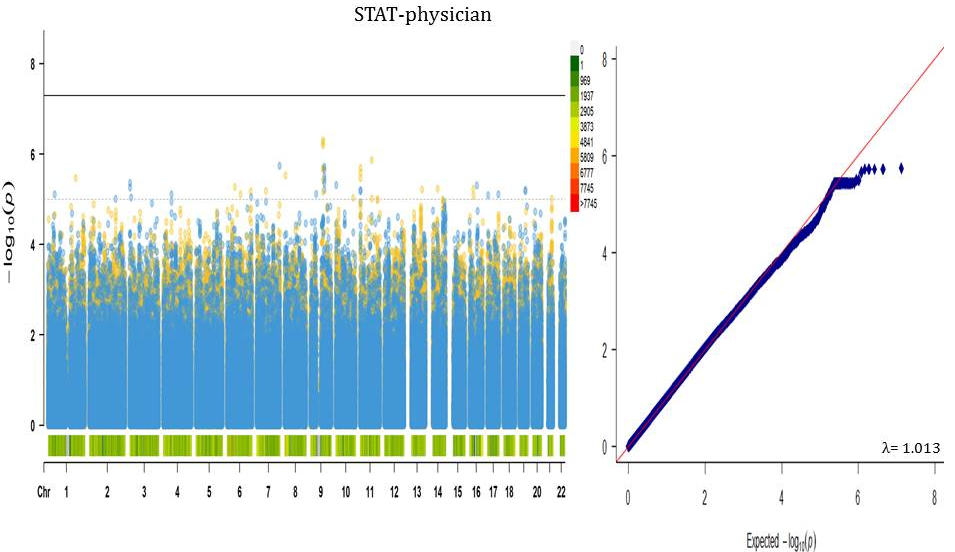


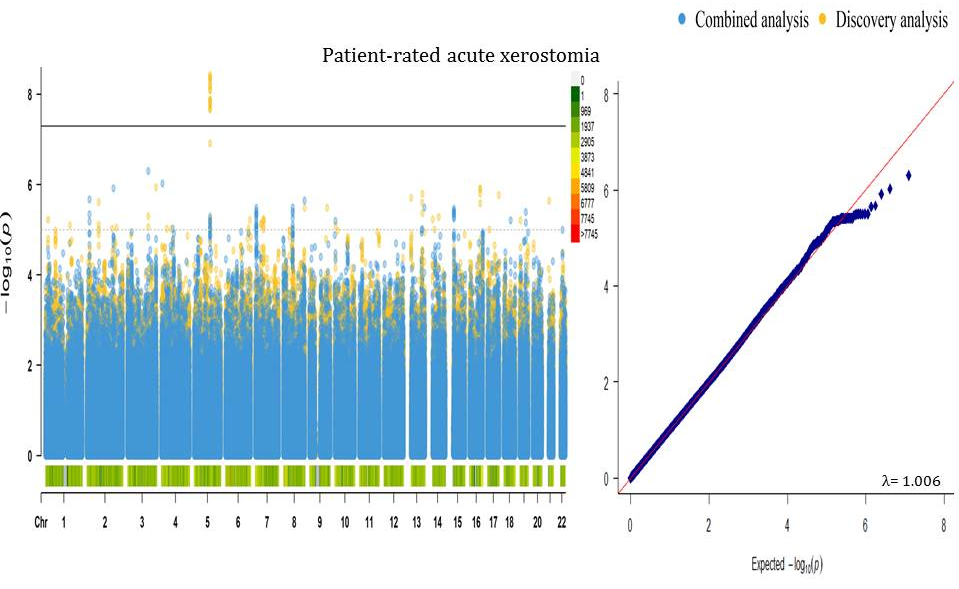


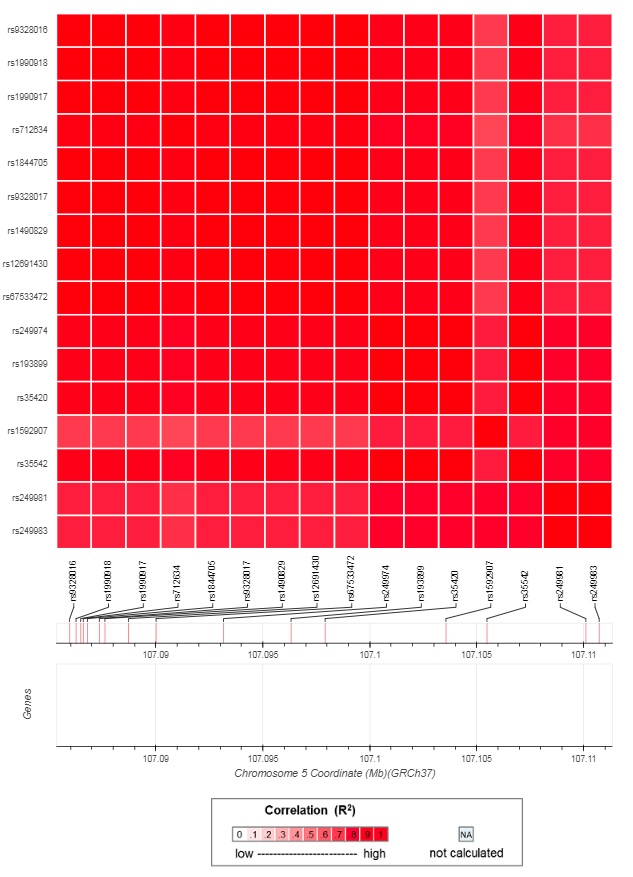


**Supplementary Figure S4:** Linkage disequilibrium (LD) plot for the genome wide significant block on chromosome 5 which contains 16 SNPs associated with patient rated toxicity xerostomia in HNC patients in the discovery cohort. The *X* axis shows location in the genome. The *Y* axis shows the rs-number of associated SNPs. Colour of cells coded according to strength of the linkage disequilibrium (r^2^ value) as indicated by the legends.

**References**

1. National Cancer Institute. Common terminology criteria for adverse events (2009). 2014;2009. Available from: http://evs.nci.nih.gov/ftp1/CTCAE/CTCAE_4.03_2010-06-14_QuickReference_5x7.pdf

2. Scott NW, Fayers PM, Aaronson NK, Bottomley A, De Graeff A, Groenvold M, et al. The use of differential item functioning analyses to identify cultural differences in responses to the EORTC QLQ-C30. Qual Life Res. 2007;16:115-129.

3. Price AL, Patterson NJ, Plenge RM, Weinblatt ME, Shadick NA, Reich D. Principal components analysis corrects for stratification in genome-wide association studies. Nat Genet. 2006;38(8):904–9.

4. Barnett GC, West CML, Coles CE, Pharoah PDP, Talbot CJ, Elliott RM, et al. Standardized total average toxicity score: A scale- and grade-independent measure of late radiotherapy toxicity to facilitate pooling of data from different studies. Int J Radiat Oncol Biol Phys. 2012;82(3):1065-74.

5. Purcell S, Neale B, Todd-Brown K, Thomas L, Ferreira M, D B, et al. PLINK: a tool set for whole-genome association and population-based linkage analyses. Am J Hum Genet. 2007;81(3):559-75.

6. Turner S. qqman: an R package for visualizing GWAS results using Q-Q and manhattan plots. J Open Source Softw. 2018;3(25),731.

7. Wei S, Li C, Yin Z, Wen J, Meng H, Xue L, et al. Histone methylation in DNA repair and clinical practice: New findings during the past 5-years. J Cancer. 2018;9(12):2072–81.

8. Toulany M. Targeting DNA double-strand break repair pathways to improve radiotherapy response. Genes (Basel). 2019;10(1):1–20.

9. Moshous D, Callebaut I, De Chasseval R, Corneo B, Cavazzana-Calvo M, Le Deist F, et al. Artemis, a novel DNA double-strand break repair/V(D)J recombination protein, is mutated in human severe combined immune deficiency. Cell. 2001;105(2):177–86.

10. Hurov KE, Cotta-Ramusino C, Elledge SJ. A genetic screen identifies the Triple T complex required for DNA damage signaling and ATM and ATR stability. Genes Dev. 2010;24(17):1939–50.

11. Makovski A, Yaffe E, Shpungin S, Nir U. Down-regulation of Fer induces ROS levels accompanied by ATM and p53 activation in colon carcinoma cells. Cell Signal [Internet]. 2012;24(7):1369–74.

12. Andreassen CN, Rosenstein BS, Kerns SL, Ostrer H, De Ruysscher D, Cesaretti JA, et al. Individual patient data meta-analysis shows a significant association between the ATM rs1801516 SNP and toxicity after radiotherapy in 5456 breast and prostate cancer patients. Radiother Oncol. 2016;121(3):431–9.
